# Efficient Nash Equilibrium Resource Allocation Based on Game Theory Mechanism in Cloud Computing by Using Auction

**DOI:** 10.1371/journal.pone.0138424

**Published:** 2015-10-02

**Authors:** Amin Nezarat, GH Dastghaibifard

**Affiliations:** 1 Department of Computer Engineering, Payame Noor University, Yazd, Iran; 2 Department of Computer Engineering, Shiraz University, Shiraz, Iran; Tianjin University of Technology, CHINA

## Abstract

One of the most complex issues in the cloud computing environment is the problem of resource allocation so that, on one hand, the cloud provider expects the most profitability and, on the other hand, users also expect to have the best resources at their disposal considering the budget constraints and time. In most previous work conducted, heuristic and evolutionary approaches have been used to solve this problem. Nevertheless, since the nature of this environment is based on economic methods, using such methods can decrease response time and reducing the complexity of the problem. In this paper, an auction-based method is proposed which determines the auction winner by applying game theory mechanism and holding a repetitive game with incomplete information in a non-cooperative environment. In this method, users calculate suitable price bid with their objective function during several round and repetitions and send it to the auctioneer; and the auctioneer chooses the winning player based the suggested utility function. In the proposed method, the end point of the game is the Nash equilibrium point where players are no longer inclined to alter their bid for that resource and the final bid also satisfies the auctioneer’s utility function. To prove the response space convexity, the Lagrange method is used and the proposed model is simulated in the cloudsim and the results are compared with previous work. At the end, it is concluded that this method converges to a response in a shorter time, provides the lowest service level agreement violations and the most utility to the provider.

## Introduction

Cloud computing is a model for the empowerment of comprehensive, convenient and demand-oriented network access to a series of share and configurable computing resources such as networks, service providers, storage space, application and other services, which can be supplied quickly and made final and usable with the least attempt and contact with the providing manager [1, 2]. The approach followed in Cloud Computing services consists of paying a certain amount for the demanded resources and providing the resources for the agreed time period. This method is currently utilized by famous cloud providers including Amazon EC2 as resource calculation per hour usage of CPU (pay as you use) [1]. Although this method is one of the most basic methods of selling commodity or services, it prevents complex interaction between the buyer and the cloud provider. One of the drawbacks of this method is that at the time of high system workloads, a job demanded by a customer must wait a long time in order for the workload to become less and the needed resources to be allocated. This strategy is free of the priority of task execution, whereas the customer owner of this job can raise their priority by bidding more money for it and obtain the needed resources. The current methods of resource allocation such as FIFO, Round-Robin, etc., used in clouds, conduct a type of unfair allocation without considering task priority between jobs.

In most cloud services, performing task scheduling with respect to these conditions and also considering the said requirements in the service level agreement is a complex task. Ordinarily, cloud providers agree on some conditions as their obligations in the provision of quality services at the time of resource allocation to customers; therefore, any resource allocations to jobs and or transferring jobs from one resource to another resource must be conducted by considering the agreed Quality of Service (QoS)[2]. For instance, if a resource is allocated to one job, no other job can obtain that resource with a higher price until the time of implementing the allocated task and, moreover, at the time of assessing the applicant’s suggestions for resource allocation, considering these parameters seems necessary to guarantee the intended quality. Some issues like deadline and maximum budget of the customer are among them. Other parameters can also be placed in this discussion including the network bandwidth for tasks which need input file and or tasks which comprise a number of subtasks (workflow).

Customers are interested in their tasks being completed in the shortest time possible and with the least cost which a cloud provider must receive. On the other hand, the cloud provider also tends to maximize the utilization of its resources and enhance its profitability, which are in contrast with each other and are not in alignment with traditional methods of resource allocation and scheduling mechanism. Since providing service in clouds is a kind of product in the supply chain, service scheduling can be divided into two categories:

1. User-Level
2. System-Level

In the system-level scheduling, solving the problem of resource management within data center is considered. From the customer’s view, a data center is an integrated system offering services to users; however, data centers are a combination of a great number of physical machines serving integratedly. After receiving a great number of tasks from different users and allocating them to physical machines, performance of a data center is influenced. In order to control and improve utilization of the system, numerous instances such as the simultaneous execution of processes, resource sharing, debugging, etc. Must be included [2, 3, 4, 5, 6]. This type of scheduling at the cloud data center level is also called resource provisioning. The focus of this study is on the subject of resource allocation. In a smaller grouping, resource scheduling and their allocation to tasks in the cloud provider can be divided into two groups [2, 6, 7, 8, 9, 10, 11, 12, 13].

- Economic models for resource-provisioning (from the user's perspective)
- Heuristic models for task-execution scheduling (from the system’s perspective)

Economic models for resource-provisioning is implementable in the format of several strategies among which the following can be mentioned:

1. **Market-based strategies include**
  - Commodity model: equivalent resource pricings
  - Posted price model: various pricings for different usage hours and methods
  - Bargaining model: initial pricing by the provider and bargaining until gaining agreed price
2. **Auction-based strategies include**
  - Number of participants: choosing auction type (selling, buying, double, etc.) based on the number of participants (bidders)
  - Based on information transparency: choosing method of presenting information to other participants such as fully transparent method, sealed method, etc.
  - Combinatorial auction method: offering bids as a combination of several bids for types of resources, as an instance, a customer offers a bid for a combination of Network, Storage, RAM, CPU.

**Fig 1:**
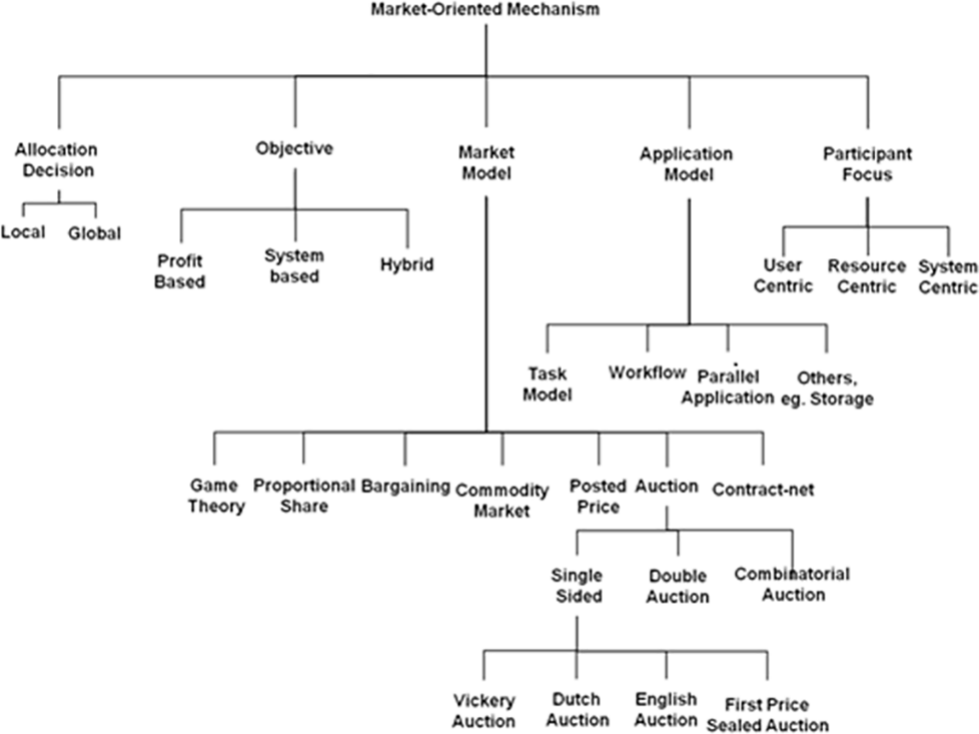
Categorizing types of resource scheduling methods in grid and economic grid [14].

In a research conducted by Buyya et al. [14], different types of economic based scheduling methods used in economic grid and grid environments are categorized according to Fig 1. Heuristic models for task-execution scheduling mainly include static and dynamic methods. Static methods are used when the set of tasks to be scheduled is predetermined and dynamic methods are used when tasks enter as real time [6].

### Static strategies

Of the most famous presented algorithms in this group, OLB,MET,MCT,MIN-MIN,MAX-MIN, GA, SA, Tabu,A*, etc. can be pointed out. Based on the conducted evaluation when the machines are compatible, GA algorithm provides the best response and MET the worst response. And for incompatible machines, A*,GA the best solution and OLB the worst. In general, Min-Min, A*, GA can be introduced as the most trusted methods in the provision of the least Makespan.

### Dynamic strategies

Such algorithms can be put in two groups of Online and Batch; in the Online mode, all tasks are scheduled as real time and each task is deposited to one of the resources after being received by the scheduler; in the Batch mode, tasks are scheduled categorically and groups are deposited to resources with a short interval.

- **Online-Mode:** KPB, SA, MCT, MET, OLB algorithms are placed in this group.
- **Batch-Mode:** Suffrage, Max-min, Min-Min algorithms are included in this category.

Amazon has had numerous innovations as one of the pioneers of cloud computing in the discussion of resource allocation. This provider has divided the supplied resources into three instances each of which is usable for a certain need.

- **The first instance and customary of Amazon is On-Flat**: In this instance, evidence of the notion of pay as you go, for hourly resource rent, a fixed pricing has been conducted and users can purchase an hour to start and, in continuance, if needed to rent more time, pay the cost of the next hour again and still have the resource at their disposal.
- **The second instance is On-Demand**: In this instance, pricing has been done on the grounds that the user rents the resource for a longer period of time right from the start; therefore, the price of renting this instance per hour is less than the previous one. In this instance, the user must be committed to paying all hours of the rented resource.
- **The third instance is On-Spot**: In this instance, Amazon reprices its additional resources, not currently rented. Amazon’s pricing model for this instance has not been provided based on minimum costs and acquisition of profitability through resources. The process of pricing these resources occurs once every hour and users applying for these resources must offer bid for their intended resources; if the offered bid is more than the base price, the resource is given to them for a period of one hour with Amazon’s base price. At the end of one hour, the new pricing is renewed and if the user’s previous bid is more than the new price, the resource rent continues for another hour until the user’s bid goes below Amazon’s pricing. If the user’s bid is less than Amazon’s new price, the task is cut without any warning and resources are taken back from the user.

In this research, inspired by Amazon’s On-Spot instance, the subject of resource allocation from user’s perspective is considered, in accordance with the instances mentioned and using auction economic method and designing an executive mechanism based on the game theory, a multi-agent environment is presented for the expansion of the cloud provider’s utility, on the one hand, and the user’s required resource allocation by considering time constraints and the user’s budget, on the other hand. In the proposed model, each agent seeks the maximization of their expected utility and the cloud provider as nature intends to maximize the utilization of cloud, in addition to maintaining the system in the Nash equilibrium mode; each agent represents a customer and is also responsible for announcing the start and end of the game and choosing the winner of the auction and calculating the provider’s utility.

## Related Works

Since market-based methods have been used in numerous researches, in this section, we review them in three subcategories, public, grid computing and cloud computing.

### In public environment

One of the oldest usages of the auction model in computer computing, dates back to computing time allocation of a mini-computer in Harvard University [15]. In this auction model, a smart “financial bid” was offered by the user to attend the auction in order to take computing time. Gagliano [16] has also studied the subject of computing resources through auction; he tried to somewhat inject smartness into tasks so as to be able to calculate a suitable bid to take resources. In this method, in order to offer each bid, the information of previous bids has also been used. Economic mechanisms are introduced as one of the main approaches of in resource management systems such as agents [1], telecommunication networks [17], data mining [18], cluster computing [19] and grid computing [20]. In these systems, numerous management instances, including bandwidth price, congestion control of TCP, content delivery and routing, are investigated and researched. Market traditional models are mainly summarized in pricing and transmission modes, among which commodity models, contract, lending and simple auction methods can be named. These models each have strengths and weaknesses and are suitable for a certain situation. Stuer [21] prefers the commodity model, since he believes that this model leads to equilibrium in supply and demand. Stanford [22] has designed architecture based on the contract method in which a dynamic pricing model exists based on the clash of demands mechanism. In this model, certain systems are designed and employed by the system in order for the price to reach a fixed point. The lending model [23] has been considered as another strategy. In this model, the two-sided cooperation of resources has been presented so that one side, instead of receiving money, obtains a resource on the other side. Additionally, numerous auction-based methods are extensively used in resource management including bid-wined and bid-shared.

### In Grid Computing

Auction has been used, to a great extent, in resource allocation and scheduling in the grid computing. Wolski et al. [24] have compared auction and commodity market models to achieve a sustainable price and balancing point. Gomoluch and Schroeder [25] have simulated a two-sided auction protocol to allocate resources in grids. They have demonstrated that the proposed method of their research has a better performance than the famous Round-Robin method. Gray et al. [14] have designed a meta scheduler based on two-sided auction which works in the grid environments. The conducted comparison by them shows that their proposed auction method, in addition to increasing user utility and system performance, offers better responses compared to the usual meta schedulers. Das and Grosu [26] have presented a combinatorial auction model to allocate resources in grid so that, in their model, various grid providers can supply their computing resources. An “auctioneer” collects different information on the supplied resources and implements a combinatorial auction mechanism to allocate resources, such that users offer their bids in order to obtain a series of resources. Dash et al. [27] formulated a scheduling mechanism of tasks in the grid environment. They considered an optimization problem with the objective of reducing the system’s whole price; nevertheless, in this model, selfish providers might attempt to present an erroneous report of the price parameters and their capacity rate if it leads to the expansion of their profit. They offered a decentralize as well as central mechanism to solve this problem. In another study conducted by Grosu [28], a system architecture has been presented for the problem of compatible resource allocation in the grid environment. The proposed architecture allows both groups of users and providers to participate in the process of resource allocation and pricing with different mechanisms.

Galstyan [8] has proposed a small supplied algorithm to allocate resources in grid environments. In this algorithm, agents that use a certain resource are rewarded if their number does not violate the resource capacity and, otherwise, are punished. Consequently, a system can use all resources by means of adjusting their capacity. The limitation of this algorithm is in the fact that few agents can be active in that. Berdin [9] has developed a non-centric debate strategy in auction for divisible resources. The amount of budget constraint is determined for mobile agents and they try to do the task by planning the available price amount through a series of repeated stages. Maheswaran [10], by expanding Berdin’s results, proposed a divisible auction structure which allows auction features to be modeled as linear Gaussian in a wide range of mobile agents. He also proved that an auction contains a single Nash equilibrium point. Nash equilibrium explains how an individual can adopt a rational decision in a non-cooperative environment; as a result, this concept is used in resource allocation researches in mobile agents and grid computing. G-Commerce [29] is an example of grid management tools which uses economic computing method to allocate and control resources. In this platform, two types of market conditions are implemented, commodity market and auction. BEinGRID [30] is also an infrastructure for the execution of grid technologies in conditions of commercial scenarios. In the GridEcon [31], a commodity market platform has been presented so that users can purchase available computing resources.

### In Cloud Computing

Recently, researchers have moved towards using economic methods in cloud computing. Wang et al. [32] have used various economic methods in resource pricing in cloud. They tested the execution of the proposed model on Amazon EC2 and a proprietary infrastructure and, in the end, concluded that the pricing used in Amazon is unfair to users. Walker et al. [33] have presented a model in which the resultant profit is calculated by obtaining the storage space from cloud. CloudCmp tools [34] help users to be able to find a suitable service provider by determining their needs. Buyya et al. [35] have proposed an infrastructure for resource allocation in multiple clouds based on commodity pricing method. Altman et al. [36] have suggested a market in which resource allocation and pricing are based on bidding exchanges. In this exchange, users and service providers both offer their requirements and bids and allocation occurs in case of finding a suitable match. Risch et al. [31] have offered a cloud test environment to experiment various mechanisms. They have implemented the model proposed by Altman et al. [36] in this platform. The proposed market in the last two studies are not easily implementable due to current cloud infrastructures and available messaging interactions. As a consequence, the main focus of most researchers is on a model where a single provider and a number of users are present. Lin et al. [37], by examining economic models with statistical data analysis, have demonstrated that economic methods can supply suitable resource allocation and acceptable income for the provider if a lot of resources and users exist. As an example, Amazon EC2 service presented one of the economic methods of selling resources, called on spot, for the first time in commercial service providers. Unfortunately, due to commerciality, the pricing method used in on spot instances is not publicly accessible. Chochan et al. [38] have demonstrated in a research how to be able to increase speed in tasks of the Map/Reduce type using on spot examples. They analyzed how this model leads to performance achievement and cost reduction. Campos-Nanez et al. [39] have conducted a research in which by implementing a dynamic auction and performing different adjustments in it in public computing environment, service providers offer their bid to implement a job instead of users. In this model, the capacity of available resources in the provider is publicly visible and bidders merely offer their bids and the service is chosen with the lowest price for the new customer. Researchers have depicted that Markov perfect equilibrium point exists for the game in this model.

The subject of resource supply is also one of the issues which researchers have attempted to solve by using economic models. Dynamic supply of computing resources has been evaluated by Quiroz et al. [40]. They presented a real time non-centric method to supply VM based on workload estimation parameters. The authors in this study proposed a model to predict workload based on estimation over a long period of time. Van et al. [41] have evaluated an automatic resource management system which is VM allocation from a physical machine sample to the resource. They demonstrated that their proposed model simultaneously guarantees the service level agreement and resource utilization. Vecchiola et al. [42] have also proposed an allocation mechanism based on deadline for the execution of scientific workflow in cloud.

Kansal et al [43] have moved towards measuring the consumption of each VM separately by developing techniques of measuring energy in physical computing resources. They tried to predict and reduce the cost of central electricity supply by creating the capacity of measuring energy consumption in each VM. Meng et al. [44] have introduced a technique to find patterns from within servers’ workload so they can attempt to combine and merge virtual machines. This model packs several VMs in a smaller resource in order to maintain service quality. Chen et al. [45] have introduced various factors for the optimization of resource utilization and reduction of costs in order to reduce cloud provider cost and enhance its profit. Their solution intends to control performance and costs using utilization tuning vector from computing resources and retuning VMs at the execution time. Naik and Ghosh [46] formulated the fact that most users’ demands are more than the computing resource amount needed and by removing additional demands attempt to increase profit. In their proposed model, the highest bid is chosen as the winner and must pay the offered amount. In another research, Lynar [47] has assessed three types of bid-winner auction and obtained a considerable difference at the execution time and consumptive energy. A great number of commercial and scientific platforms use economic methods to solve the problem of resource allocation in grid and cloud. Cloudbus [48] is an infrastructure interface and a middleware to develop cloud computing programs based on economic methods. The above-mentioned frameworks refer to conceptual environments of the problem of resource allocation in cloud and grid using final equilibrium theory and optimization from user’s perspective. The subject of cooperation in computing economy merely consists of creating equilibrium between users and providers of cloud service in order to enhance utilization of resources and competition among different users has been avoided. A common flaw in most of these studies is that, in the hypothesized non-centric models, the environment is considered as non-competitive and ideal. Mobile agents perfectly understand their competitors’ information, a fact which is hardly plausible in the real world. Kwok [7] is the pioneer in using hierarchical game theory models in grid computing environments (greedily). He has used both equilibrium point method and problem optimization strategy for public modes based on utility function.

In an alternate research [12], a suitable method has been presented to allocate resources for multi-agent systems. In this method, the presence of Nash equilibrium point has been relied on. Commercial agents can improve the results of conducted allocations by means of updating their belief from environment and introduce new bids. Wei [6] has used both optimization and justice method by considering a resource allocation problem for cloud computing. He attempted to solve the problem of optimization using problem relaxation methods with the help of binary integer programming and utilized a game theory evolutionary mechanism to reduce the amount of lost performance. As was pointed out, various studies have been conducted by researchers in the field of resource allocation and supply methods in computing environments especially grid. According to the great number of decision-making parameters in cloud environment and evaluating the conducted movement process of researches, it seems that economic scheduling methods, due to beneficiaries’ two-sidedness in cloud environment and existence of financial problems between the parties, can be a suitable option for continuing researches in this field. In accordance with the proposed categorization in economy-based methods [49], six groups of resource allocation and supply algorithms have been presented so far of which auction methods and methods with auction-based pricing are at the head of the proposed models; this issue has been mentioned separately in Table 1.

**Table 1:** Comparison between various types of resource allocation economic methods [49].

Economic method type	Profit	Income	Allocation performance	Execution time
Double auction	For both sides	Medium	High	Low
Reverse auction	User	Low	Medium	Very low
Fixed and posted price auction	Service Provider	High	High	Very low
Vickrey auction	User	Low	High	Low
Combinatorial auction	For both sides	High	Very high	Very low

By evaluating different models presented in allocation in economic environments in Table 2 [50, 51, 52, 53, 54, 55, 56, 57, 58, 59], in this research, we intend to more accurately investigate the presented algorithms in auction, auction sharing, posted price, bargaining, commodity price, contractual network and game theory methods, and propose a suitable method of one or a combination of a number of these methods. According to the conducted categorization, it seems that auction-based methods, auction sharing and commodity price have had the most inclination to utilization [60,61,62,63]. The commodity price method, due to very high simplicity and faster execution, has been able to attract the attention of a number of researchers. However, this method, due to being based on commodity pricing using knowledge of the market methods and other service provider and market movement strategies, is more considered as a static pricing strategy than a dynamic resource allocation method. The auction method, due to using a parametric and fair approach offering a win-win result to both sides, has been considered by many researchers [64,65,66,67,68]. In this study, we try to provide a new model based on auction or a combination of auction with other methods, by investigating and comparing different models in this area and along with eliminating their deficiencies.

**Table 2:** Algorithms and tools based on resource allocation economic methods.

Model or algorithm title	Mechanism	Economic model group
Tycoon, Spawn, Bellagio, OCEAN, SORMA, PeerMart, G-Commerce	Auction sharing, Vickrey auction, combinatorial auction	Auction, auction sharing
Sharp, Shirako, Nimrod-G, GridEcon, Gridbus, Java Mrkaet, Mariaposa		Commodity model
GRIA, CatNets		Bargaining

## Problem Modeling

First, the problem is evaluated at the level of platform as a service, in the modeling of this problem, it is assumed that tasks are relegated to Cloud at the level of platform and the service provider needs to have information about tasks in order to be able to acquire the best combination of virtual machines for the execution of tasks from infrastructure level.

We assume that n tasks are allotted to m resources. Each user *U*_*i*_ have a number of similar interdependent *k*(*i*) tasks which need equal amount of computing. Each resource is in such a way that the sum of total cost is minimized and n relevant tasks are implemented in the least time with minimal cost. We assume matrix *a* consisting of n rows, each for one user, and m columns each for one resource. *a*_*ij*_ represents the amount of each task of user *U*_*i*_ that is assigned to *R*_*i*_, then we assume that *a*_*i*_ is the representative of i-th row of the matrix *a*. Hence, the allocation vector of *a*_*i*_ can result in: $\sum_{a_{ij}\in a_{i}}a_{ij}=k(i)$

Two matrices of *n* * *m* are derived from the *a* matrix, one is completion time matrix *T* and the other is expense matrix *E*. The *t*_*ij*_ amount of *T* matrix indicates the execution time of the tasks od user *U*_*i*_. When they are assign to *R*_*i*_ resource, since all tasks of user *u*_*i*_ are implemented in parallel and independently, the execution time of all tasks of user *u*_*i*_ equals max{*t*_*ij*_|*t*_*ij*_ ∈ *t*_*i*_} so that *t*_*i*_ is indicative of the i-th row of the *T* matrix. The *e*_*ij*_ amount of the *E* matrix represents the price paid by user *U*_*i*_ to implement its *a*_*ij*_ task on *R*_*j*_ resource. Therefore, the price for implementing the *U*_*i*_'s task equals $\sum_{j=1}^{m}e_{ij}$. In general, a contrast and trade-off exists between the execution time and cost for each of the tasks. Here we assume that all the participants have complete information of the environment and identical view of the cost and time matrix.

To create equilibrium between time and cost, two amounts of *W*_*t*_ for weighing time and *W*_*e*_ for weighing fee are hypothesized; therefore, the total cost of user *U*_*i*_ is written as follows: $w_{t}.{max}_{t_{ij}\in t_{i}}\{t_{ij}\}+w_{e}\;\sum_{j}\;e_{ij}$. And since the ultimate objective is to minimize cost and increase utility, the amount of utility for the *U*_*i*_ can be calculated as thus:

$$u_{i}(a_{i})=\frac{1}{w_{t}.{max}_{t_{ij}\in t_{i}}\{t_{ij}\}+w_{e}\;\sum_{j}\;e_{ij}}$$

In addition, we assume that

1. Resource capacity is not divisible
2. Each resource can be allocated to various tasks
3. All resources use time-sharing policy for the scheduling of tasks allocated to them at the level of the Operating System.

The ${\hat{t}}_{ij}$ is assumed as the execution time of the i task on the *R*_*j*_ resource in such a way that no other task is being implemented on that resource. It can be deduced that $t_{ij}={\hat{t}}_{ij}.\sum_{j}\;a_{ij}$ and $e_{ij}=a_{ij}.{\hat{t}}_{ij}.p_{j}$ are in place when only one task is allocated to a resource. *P*_*j*_ stands for the cost of resource per time unit in such a way that P = *P*_1_, *P*_2_, …, *P*_*m*_ is arranged ascendingly. In order to display the modeled problem, an example is investigated using this model. We assume that we have 4 resources of *R*1 – *R*4 so that the cost vector is as P = (1.2, 1.4, 1/7, 2) and we intend to allocate 4 tasks of *U*_1_ – *U*_4_ to these resources.

The execution time of tasks on resources, so that resources execute each task separately, is as follows:

$${\hat{t}}_{ij}=\left[\begin{array}{cccc} 7 & 6.3 & 5.7 & 5\\ 6 & 5.1 & 4.5 & 4\\ 5 & 4.5 & 4 & 3.5\\ 4.5 & 4 & 3.5 & 3 \end{array}\right]$$

We assume that initially user *U*_1_ chooses resources {R1, R2} and *U*_2_, resources {*R*1, *R*2, *R*3} and *U*_3_, resources {*R*1, *R*2, *R*4} and *U*_4_, resources {*R*1, *R*2, *R*3, *R*4}.

Consequently, the assignment matrix is as follows:

$$a=\left[\begin{array}{cccc} 1 & 1 & 0 & 0\\ 1 & 1 & 1 & 0\\ 1 & 1 & 0 & 1\\ 1 & 1 & 1 & 1 \end{array}\right]$$

Since the cost and time of executing all task of users on resources are jointly divided, the execution time of tasks is calculated thus:

$$t_{ij}=\left[\begin{array}{cccc} 28 & 25.2 & 0 & 0\\ 24 & 20.4 & 9 & 0\\ 20 & 18 & 0 & 7\\ 18 & 16 & 7 & 6 \end{array}\right]$$

And the cost matrix:

$$e_{ij}=\left[\begin{array}{cccc} 28*\frac{p1}{4} & 25.2*\frac{p2}{4} & 0 & 0\\ 24*\frac{p1}{4} & 20.4*\frac{p2}{4} & 9*\frac{p3}{2} & 0\\ 20*\frac{p1}{4} & 18*\frac{p2}{4} & 0 & 7*\frac{p4}{2}\\ 18*\frac{p1}{4} & 16*\frac{p2}{4} & 7*\frac{p3}{2} & 6*\frac{p4}{2} \end{array}\right]$$

$$=\left[\begin{array}{cccc} 8.4 & 8.82 & 0 & 0\\ 7.2 & 7.14 & 7.65 & 0\\ 6 & 6.3 & 0 & 7\\ 5.4 & 5.6 & 5.95 & 6 \end{array}\right]$$

In order to maintain simplicity in computations, we ignore the ratio of weight time to cost. Therefore, each of the players (users) is as follows:

$$u_{1}(a_{1})=\frac{1}{\left(28+(8.4+8.82)\right)}≅0.0221$$

$$u_{2}(a_{2})=\frac{1}{\left(24+(7.2+7.14+7.65)\right)}≅0.0217$$

$$u_{3}(a_{3})=\frac{1}{\left(20+(6+6.3+7)\right)}≅0.0255$$

$$u_{4}(a_{4})=\frac{1}{\left(18+(5.4+5.6+5.95+6)\right)}≅0.0244$$

Each of the tasks is considered as a player and the *a*_*i*_ assignment vector of each is also assumed as that player’s selective strategy. In the end, the ultimate strategy of the system’s final mode including total selective strategies by all players in the T time will be as *a* = (*a*_1_, …, *a*_*n*_)^*T*^ vector. The selective strategy of each player *i* is demonstrated with *a*_*i*_ and other competitors’ strategy is shown as *a*_−*i*_. Accordingly, (*a*_*i*_, *a*_−*i*_) stands for the selective strategy of the player *i* against other players and in case of alteration in the strategy of player *i*, any kind of adoption of other strategies is displayed as (*a*′_*i*_,*a*_−*i*_). In the end, the purpose of the problem is to solve the game G = < *s*, *a*, *u* >, in which *s* represents players, *a* their strategies and *u* their obtained utility. This game is of the type of repetitive and simultaneous game. Each player seeks maximum amount of utility and in order to optimize the solution of the problem, the following conditions must be considered:

$$Maximize\;u_{i}(a_{i})$$

$$Subject\;to\;\sum_{a_{ij}\in a_{i}}a_{ij}=k(i)$$

$$a_{ij}\geq 0$$

In which k(i) stands for all the tasks of user *U*_*i*_. One of the key parameters in obtaining utility function, is an amount called Constant Elasticity of Substitution (CES). This amount seeks to obtain a fixed amount of demand value (computational resources) and production value (virtual machines) and create an equilibrium in them. The amount of CES can be calculated as thus: $C=\left[{\left[\sum_{i=1}^{n}\alpha_{i}^{1/S}c_{i}^{(S-1)/S}\right]}^{S/(S-1)}\right]$

C is the integration value of using all resources by all players, *c*_*i*_ is each palyer’s usage of (the player’s share of resources) such as execution time, paid price, consumption bandwidth, used electricity, etc. *α*_*i*_ is also the resource share parameter which detects each resource allocation value to a task. And *s* is demand elasticity of substitution. In order to simplify, equation $r=\frac{s-1}{s}$ is considered and *C* is rewritten as follows:

$$\ln\;C=\frac{\ln\;\sum_{i=1}^{n}\left({\alpha_{i}}^{1-r}c_{i}^{r}\right)}{r}$$

And after applying the Hoptial law

$$\underset{r\to 0}{\lim}\;\ln\;c=\frac{\sum_{i=1}^{n}\alpha_{i}\;\ln\;c_{i}}{\sum_{i=1}^{n}\alpha_{i}}$$

If $\sum_{i=1}^{n}\alpha_{i}=1$, the function value will be as aconstant scale, namely that the consumption amount will grow as the consumption growth rate value of each of the resources. If each *α*_*i*_ grows 20%, then *C* will also grow 20%. If $\sum_{i=1}^{n}\alpha_{i}<1$, then the scale return rate declines or on the other hand rises. CES function is, particularly, a specific mode of Cobb Douglas function. In a general condition, if *c*_1_, *c*_2_, …, *c*_*m*_ are consumption values from m resources by the allocated tasks to them, the utility function is written as below:

$$\bar{u}(c)=\prod_{i=1}^{m}c_{i}^{\alpha_{i}}$$

Which *c* = (*c*_1_, *c*_2_, …, *c*_*m*_) and $\sum_{i=1}^{m}\alpha_{m}=\alpha$ we will have *C* ⟼ *c* ^1/*α*^ function when > 0.

$$u(c)={\bar{u}(c)}^{1/\alpha }$$

Now assuming $p_{i}=\frac{\alpha_{i}}{\alpha }$ (ratio of using each resource to all usage of resources), again we will have:

$$u(c)=\prod_{i=1}^{m}c_{i}^{p_{i}}$$

$$\sum_{i=1}^{m}p_{i}=1$$

And to obtain maximum utility, we can take logarithm of the utility function:

$$\max\sum_{i=1}^{m}\left(p_{i}\;\ln\;c_{i}\right)$$

## Choosing optimal strategy and Nash equilibrium

Nash equilibrium is a point of game where no player will gain more utility by altering their strategy, if other players do not wish to change strategy. In this game, it is assumed that all the players are rational and have normal behavior. Once again, game G is assumed, each player *i* can obtain their utility for different modes of *u*_1_(a_1_, a_2_, …, a_*n*_) strategy. It must be noted that utility is dependent on each player’s chosen strategy and other players’ strategies. A profile of $\{a_{1}^{*},a_{2}^{*},..,a_{n}^{*}\}\in a$ strategy is a Nash equilibrium point if no change of strategy by a player leads to profit increase of other players.

$$\forall i,a_{i}\in a,a_{i}\neq a_{i}^{*}\;:\;u_{i}(a_{i}^{*},a_{-i}^{*})>u_{i}(a_{i},a_{-i}^{*})$$

In order to simplify the game and reducing limitations of optimization problem solving, the following instances are considered as presuppositions of problem solving:

1. The execution time for each of the tasks allocated to resources is predetermined. Various methods exist to estimate this time [9, 14].
2. tasks are interdependent and can interact with each other. The amount of consumption bandwidth is not considered in every interaction.

Most of scientific problems which are implemented in parallel satisfy these two terms. Moreover, if a task does not include subtasks and is implemented independently, it is sufficient to merely consider the sweep interactive time between subtasks as zero for that task.

## Objective function definition

The best objective function can be based on prices. For this purpose, the weighing sum of time and cost can be obtained [19]. Each time unit is recorded as a fixed amount of cost. In this problem, each time unit is considered the equivalent of 100 monetary unit and the ratio *w*_*t*_:*w*_*e*_ = 1 is assumed. Each player can consider a deadline, displayed with *T*_0_, and a maximum budget amount, shown with *E*_0_.

First, we assume that each resource has a certain execution capacity of tasks which is shown with *c* = (*c*_1_, *c*_2_, …, *c*_*m*_) and the amount of execution speed of each task on each resource is demonstrated through equation *e*_*ij*_. Once again, the *e*_*ij*_ matrix, representing the cost of executing the *i* task on the resource, we assume that Θ_*j*_ is the sum of task bids for the resource as the price of one resource at that instant and obtain as $\Theta_{j}=\sum_{i=1}^{n}e_{ij}$. And $\theta_{j}^{-i}=\sum_{k\neq i}^{n}e_{kj}$ is assumed as the sum of bids by other competitors for the resource *j*. It can be perceived from the above equations that the share of each task from the resource is calculable from equation. The spent time for the execution of task *i* on the resource j is defined as thus:

$$t_{j}^{i}=\frac{t_{ij}}{c_{j}x_{j}^{i}}=w_{j}^{i}+w_{j}^{i}\frac{\theta_{j}^{-i}}{e_{ij}}$$

And the final execution cost on the resource j is as thus:

$$e_{j}^{i}=e_{ij}*t_{ij}=w_{j}^{i}\theta_{j}^{-i}+w_{j}^{i}e_{ij}$$

Now, for each player to choose their best strategy, an amount must be considered as their payoff. As an example, if $t_{j}^{i}$ is assumed as the payoff, all players tend to have the most speed in task execution; therefore, the least payoff is for the best choice. On the other hand, if the price is considered as payoff ($e_{j}^{i}$), the most cost constantly dominates alternate strategies (dominated strategy) and the most $e_{j}^{i}$ dominates other competitors. Consequently, the best solution in choosing payoff is the combination of these two limitations together. According to the constant elasticity of substitution (CES) function [13], mentioned before, the players’ payoff is altered as below:

$$\varphi =\frac{\left(\rho_{e}\;\ln\;\sum_{j=1}^{m}e_{j}^{i}+\rho_{t}\;\ln\;\sum_{j=1}^{m}t_{j}^{i}\right)}{\rho_{e}+\rho_{t}}$$

So that and are, respectively, time demand elasticity output and price. In Table 3, the used parameters are presented in the model.

**Table 3:** Used parameters in the proposed model.

Final execution cost on resource j	$e_{j}^{i}$
Time taken to implement task i on resource j	$t_{j}^{i}$
Maximum budget amount	*E*_0_
Deadline	*T*_0_
Execution speed of each task on each resource	$w_{j}^{i}$
Execution cost of task i on each resource j	*e*_*ij*_
Total of all bids of tasks for resource j	Θ_*j*_
Total bids of other players for resource j	$\theta_{j}^{-i}$
Time demand elasticity output	*ρ*_*t*_
Cost demand elasticity output	*ρ*_*e*_
Estimation of total bids by other players	${\bar{\theta }}_{j}^{-i}$
Current bid i for resource k	*e*_*ik*_
Bids vector of player i	*E*(*e* ^*i*^)
Bid of player i in round m for resource k	$e_{ik}^{m}$
Equilibrium bid	$e_{ik}^{*}$
Optimum equilibrium amount of competitors	$\theta_{k}^{-i*}$

(the cost ratio of each player to other players) which, in fact, this weighing coefficient demonstrates the significance of cost or time parameter. Since the objective of all players is to minimize time and cost, then the optimal objective function of a player *i* is

minφ

$$subject\;to\;\sum_{j=1}^{m}e_{j}^{i}\leq E_{0\;},\;\sum_{j=1}^{m}t_{j}^{i}\leq T_{0}$$

By Lagrangian definition, we obtain Hamilton equation:

$$L=\varphi +\lambda_{e}^{i}\left(\sum_{j=1}^{m}e_{j}^{i}-E_{0}\right)+\lambda_{t}^{i}\left(\sum_{j=1}^{m}t_{j}^{i}-T_{0}\right)$$

By replacing parameters $t_{j}^{i}$ and $e_{j}^{i}$, function is made by three variables *e*_*ij*_, $\lambda_{e}^{i}$, $\lambda_{t}^{i}$, and by its derivation, ratios *e*_*ij*_, $\lambda_{e}^{i}$, $\lambda_{t}^{i}$ are, respectively, obtained to the amount bid by player *i* for resource *j*.

1 – Therefore we will have:

$$\frac{\partial L}{\partial e_{ij}}=\frac{\rho_{e}}{\rho_{e}+\rho_{t}}.\frac{w_{j}^{i}}{\sum e_{ij}}-\frac{\rho_{t}}{\rho_{e}+\rho_{t}}.\frac{w_{j}^{i}\theta_{j}^{-i}}{\sum t_{j}^{i}e_{ij}^{2}}+\lambda_{e}^{i}w_{j}^{i}-\lambda_{t}^{i}\frac{w_{j}^{i}\theta_{j}^{-i}}{e_{ij}^{2}}=0$$

By simplifying the above equation and applying derivation, ultimately we will have: $e_{ik}=e_{ij}\sqrt{\frac{\theta_{k}^{-i}}{\theta_{j}^{-i}}}$

2 – Partial derivative with respect to $\lambda_{e}^{i}$ (price parameter)

$$e_{ik}=\frac{E_{0}-\sum_{j=1}^{k-1}w_{j}^{i}\theta_{j}^{-i}-w_{k}^{i}\theta_{k}^{-i}-\sum_{j=k+1}^{m}w_{j}^{i}{\bar{\theta }}_{j}^{-i}}{\sum_{j=1}^{k-1}w_{j}^{i}\sqrt{\frac{\theta_{j}^{-i}}{\theta_{k}^{-i}}}+w_{k}^{i}+\sum_{j=k+1}^{m}w_{j}^{i}\sqrt{\frac{{\bar{\theta }}_{j}^{-i}}{\theta_{k}^{-i}}}}$$

3 – Partial derivative with respect to $\lambda_{t}^{i}$ (time parameter)

$$e_{ik}=\frac{\sum_{j=1}^{k-1}w_{j}^{i}\sqrt{\theta_{j}^{-i}\theta_{k}^{-i}}+w_{k}^{i}\theta_{k}^{-i}+\sum_{j=k+1}^{m}w_{j}^{i}\sqrt{{\bar{\theta }}_{j}^{-i}\theta_{k}^{-i}}}{T_{0}-\sum_{j=1}^{m}w_{j}^{i}}$$

*T*_0_ and *E*_0_ are, respectively, time and price limit of player *i* and ${\bar{\theta }}_{j}^{-i}$ also represents estimation of sum total of bids by other competitors. Each of formulas 2 and 3 indicate the effect of price and time on the bid of player *i*. Both these equations demonstrate that the current bid *e*_*ik*_ is obtained with respect to previous bids by competitors (j<k) $\theta_{j}^{-i}$, present $\theta_{k}^{-i}$ and future $\theta_{j}^{-i}$(j>k).

$$\left[\theta_{1}^{-i},\ldots ,\theta_{k}^{-i},{\bar{\theta }}_{k+1}^{-i},\ldots ,{\bar{\theta }}_{m}^{-i}\right]$$

Here, in order to simplify problem solving, it is assumed that others remain constant in the network, consequently, the game is modeled as a static game with complete information. However, since such matter is farfetched in the real world and competitors try to acquire the most utility, in practice, our information of the environment is not complete hence an estimation must be obtained of sum total of bids by other competitors compared with their past.

$$\left[\theta_{1}^{-i},\ldots ,\theta_{k}^{-i},{\bar{\theta }}_{k+1}^{-i},\ldots ,{\bar{\theta }}_{m}^{-i}\right]$$

In which, ${\bar{\theta }}_{k+1}^{-i},\ldots ,{\bar{\theta }}_{m}^{-i}$ is an estimation of bids by competitors in future.

## Estimation of parameters

The presence of Nash equilibrium in the game with complete information has been proved by Berdin [9]. Nevertheless, the new problem is when buyers do not intend to publicly display their prices. In this environment, this question is addressed: how do players become aware of each other’s prices and or, in other terms, estimate them. Since in the auction game explained before, we have the history of various round of the game Θ_1_, …, Θ_*k*−1_, we can use them to estimate the price of next round.

In probability science, Bayesian theory indicate that the likelihood a hypothesis occurring depends on the observation of given causes in the state space. Distribution of future state can be obtained from past *P*(Θ) and its similarity function *P*(Θ|Θ_*k*_), as follows:

$$P(\Theta |\Theta_{k})=\frac{P(\Theta_{k}|\Theta )\;P(\Theta )}{\int P(\Theta_{k}|\Theta )P(\Theta )d\Theta }$$

To calculate *P*(Θ|Θ_*k*_), Bayesian learning mechanism can be utilized. Using Bayesian learning, the closest amount of resource price can be predicted and next bids can be estimated as thus.

$${\bar{\theta }}_{k+1}^{-i}=E(\Theta |\Theta_{k})-E(e^{i})\ldots \ldots .$$

$${\bar{\theta }}_{m}^{-i}=E(\Theta |\Theta_{m-1})-E(e^{i})$$

In which, *E*(*e* ^*i*^) is the vector of bids by player *i*. The three parameters $\alpha_{k}^{i}$, $\beta_{k}^{i}$, and $\gamma_{k}^{i}$ are defined as knowledge from other competitors:

$$\alpha_{k}^{i}=\sum_{j=1}^{k-1}w_{j}^{i}\theta_{j}^{-i}+\sum_{j=k+1}^{m}w_{j}^{i}{\bar{\theta }}_{j}^{-i}$$

$$\beta_{k}^{i}=\sum_{j=1}^{k-1}w_{j}^{i}\sqrt{\theta_{j}^{-i}}+\sum_{j=k+1}^{m}w_{j}^{i}\sqrt{{\bar{\theta }}_{j}^{-i}}$$

$$\gamma_{k}^{i}=\sum_{j=1}^{k-1}w_{j}^{i}+\sum_{j=k+1}^{m}w_{j}^{i}$$

By replacing $\theta_{j}^{-i}$ in $F_{k}^{i}(\Theta_{k})$ in equation 2, function $F_{k}^{i}(\Theta_{k})$ is obtained as follows:

$$F_{k}^{i}(\Theta_{k})=\frac{(E_{0}-\alpha_{k}^{i}-w_{j}^{i}\Theta_{k}{)}^{2}}{2(\beta_{k}^{i}{)}^{2}}(\sqrt{1+\frac{4(\beta_{k}^{i}{)}^{2}\Theta_{k}}{(E_{0}-\alpha_{k}^{i}-w_{j}^{i}\Theta_{k}{)}^{2}}}-1)$$

**Fig 2:**
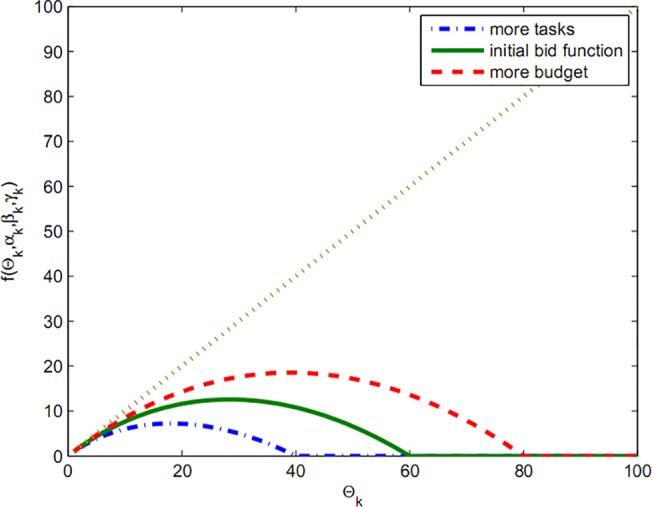
Bid with budget limit.

**Fig 3:**
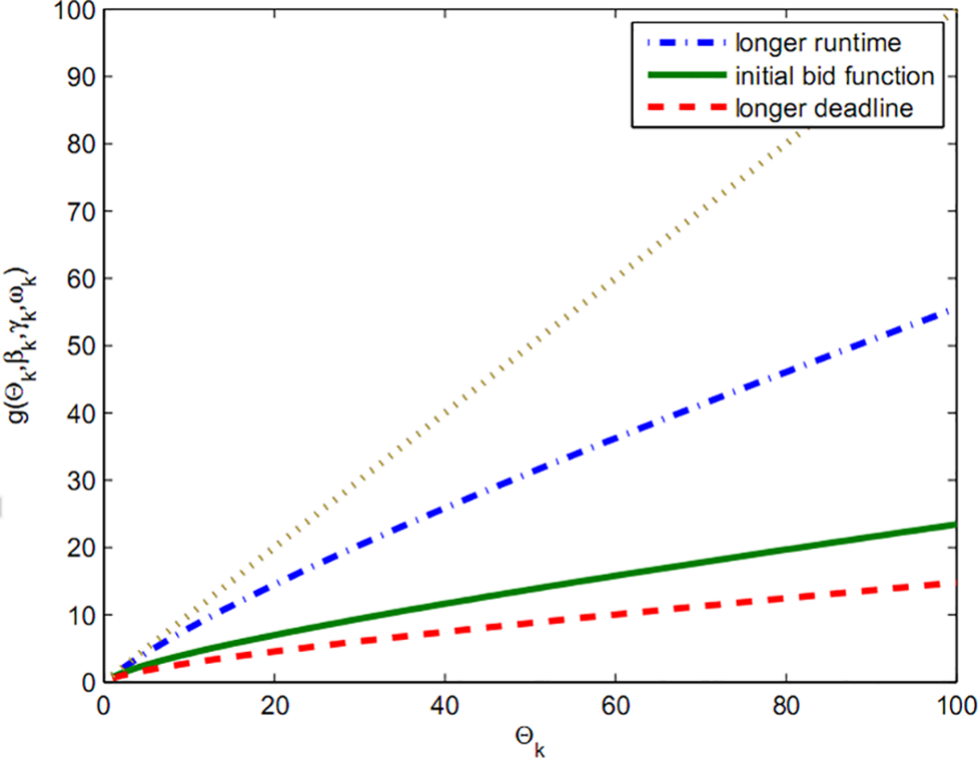
Bid with deadline.

In fact, function $F_{k}^{i}(\Theta_{k})$ demonstrates that the rate of dependence of bids is not merely on the budget and the offered amount can also change in the next stage by alteration in workload. As seen in chart of Figs 2 and 3, a rich player can gain more resources by presenting a big bid and, on the other hand, player with high workload must wait to gain cheaper resources.

By replacing $\theta_{j}^{-i}$ with Θ_*k*_ – *e*_*ik*_ in equation 3, function $g_{k}^{i}(\Theta_{k})$ is obtained which formulates the bid according to deadline:

$$g_{k}^{i}(\Theta_{k})=\frac{w_{k}^{i}}{T_{0}-\gamma_{k}^{i}}\Theta_{k}+\frac{\sqrt{{\left(\beta_{k}^{i}\right)}^{2}+4{\left(\beta_{k}^{i}\right)}^{2}(T_{0}-\gamma_{k}^{i})(T_{0}-\gamma_{k}^{i}-w_{k}^{i})\Theta_{k}}}{{2(T_{0}-\gamma_{k}^{i})}^{2}}-\frac{{\left(\beta_{k}^{i}\right)}^{2}}{{2(T_{0}-\gamma_{k}^{i})}^{2}}$$

This ascending uniformity equality function depends on and shows that if we do not include budget limit to the bid amount, it can grow indefinitely.

**Fig 4:**
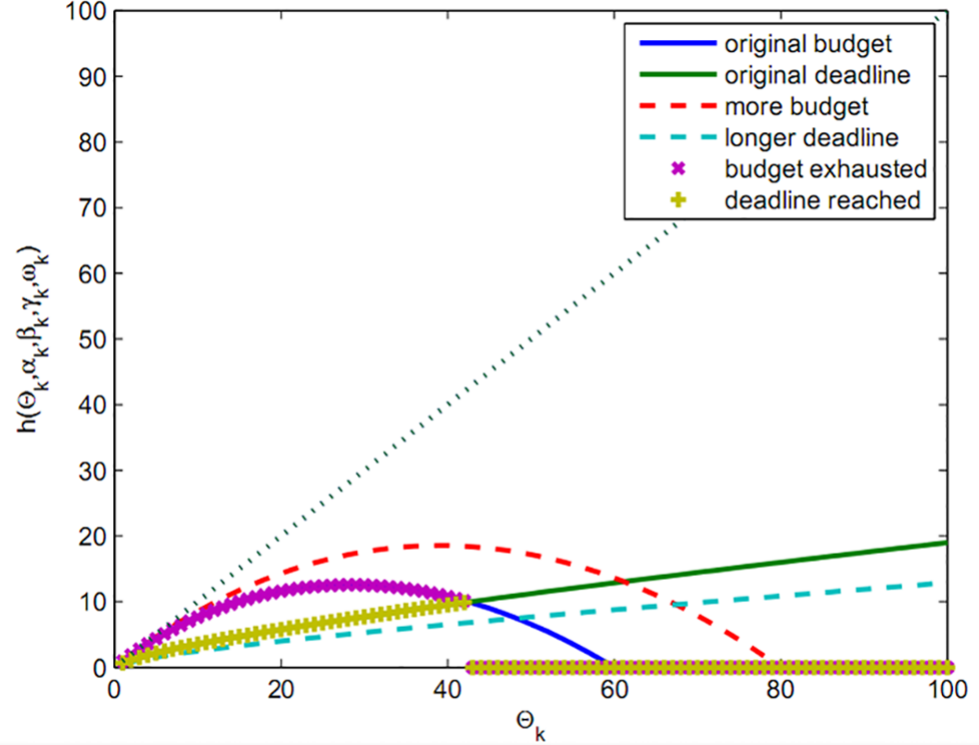
Bid with budget limit and deadline.

In Fig 4, the bid functions, when both budget limit and deadline are included, are compared.

In Fig 4, the intersecting curve is included as a new function named $h_{k}^{i}(\Theta_{k})$ (the calculated objective function the players’ bid for participating in the auction), which is based on budget limit and deadline and is written as below:

hki(Θk)={Fki(Θk):Fki(Θk)≥gki(Θk)0:Fki(Θk)<gki(Θk)

## Equilibrium price

In order to achieve an equilibrium point, in bids offered by different players, we act as follows.

First, players who need resource *k*, calculate and offer their initial bid: $\Theta_{k}^{\left(1\right)}=\sum_{N}e_{ij}$

In the first stage, the price that the players wish to pay for a part of resource, is calculated by bid function $h_{k}^{i}\left(\Theta_{k}^{\left(1\right)}\right)$. in general, we have: $e_{ik}^{m}=h_{k}^{i}\left(\Theta_{k}^{\left(m\right)}\right)$. So that *m* shows the proposal stage. Therefore, the amount that the seller intend to receive from all *N* players, is really $\Theta_{k}^{\left(m+1\right)}=\sum_{N}h_{k}^{i}(\Theta_{k}^{\left(m\right)})$.

As a consequence, part of resource owned is as $x_{k}^{i(m)}=\frac{e_{ik}^{m}}{\Theta_{k}^{\left(m+1\right)}}$

If each of the players is not satisfied with the performed allocation, due to the low resource or high price, the repetitions continue and players can optimize the bids in next stages and if all players are satisfied, the price flows: $\Theta_{k}^{\left(m+1\right)}=\Theta_{k}^{\left(m\right)}$. Therefore, the resource price in the amount $\Theta_{k}^{\left(m+1\right)}$ is consented by all players and we have reached the equilibrium price. Since, in the game theory, Nash equilibrium point occurs when none of the players intends to alter their game, thus

$$e_{ik}^{*}=\max\;x(e_{ik},\theta_{k}^{-i*})$$

**Fig 5:**
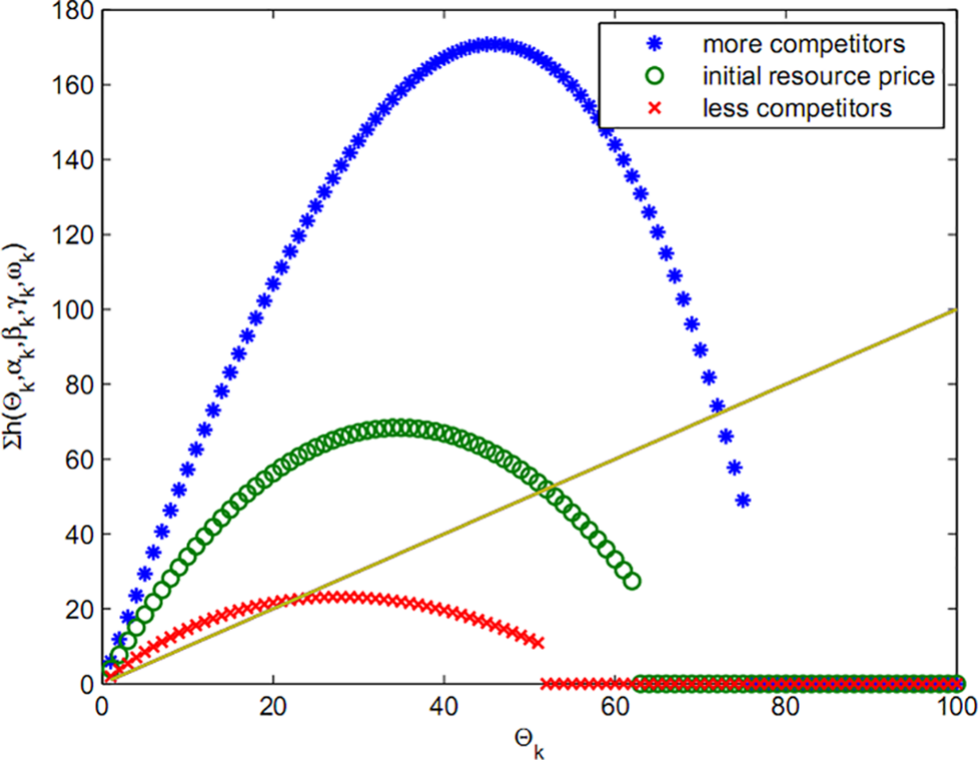
Resource equilibrium price in price limit and deadline.

So that $e_{ik}^{*}$ is equilibrium bid and $\theta_{k}^{-i*}=\Theta_{k}^{*}-e_{ik}^{*}$ competitors’ equilibrium optimum amount. When demand is greater than supply, $\sum_{N}x_{k}^{i}>1$, players are motivated to offer more price to achieve more share of resources; consequently, resource price increases. The expansion of resource price leads to the reduction of $x_{k}^{i}$ until $\sum_{N}x_{k}^{i}$ reduces to one will (a number of competitors withdraw from participating in the competition, due to lack of ability to offer higher bid). Conversely, this situation $\sum_{N}x_{k}^{i}<1$ also applies. Fig 5 demonstrates resource price equilibrium in dynamic conditions for a time when the distribution of players’ bids is similar.

## Experimentation

Since the execution of resource allocation algorithm will be very expensive and time-consuming in cloud computing real environments, therefore, the execution of this algorithm was done in cloudsim simulator [48]. First, the auction was sequentially implemented based on different systems and, afterwards, a simulation was simultaneously conducted from posted mode of proposals by different users. The possibility arrival rate of offer distribution is considered as parameters and resources can also be connected to the center or leave it freely. In simulation, four entities are considered. The CIS storage, a database of whole cloud, is used for recording between users’ needs and available resources in the datacenter. The datacenter is responsible for the integration between hardware, databank of storage tools, applied software and operating systems for the construction of a resource pool and their virtual offering to users. Next entity is users or the players who intend to execute a set of independent tasks and are ready to pay a price in order to obtain their required resources to execute tasks on those resources. These players offer their bids based on economic capacity and priority of tasks according to the proposed formula in the model. The auctioneer is a medium for the creation, management and execution of an economic competitive environment.

The auctioneer tries to obtain a monetary equilibrium point for a non-cooperative game among players based on a predetermined decision-making function of laws. At the beginning, the database prepares available resources and registers their distribution information inside CIS. Furthermore, users demanding task execution announce to the auctioneer and are placed in the queue according to their request time. In a regular cycle, the auctioneer collects the set of information and posts the demands after decision-making to datacenters in order to construct relevant virtual machines to the number needed for task execution. At each stage of auction process, each of the users post their demand including local time, processors’ power and intended bid. The auctioneer receives all bids and informs all the players of the sum total of received bids. In a game with incomplete information, Cloud users are merely aware of their bid and total bids. They dynamically forecast resource future price and update their information on competitors $\left[\theta_{1}^{-i},\ldots ,\theta_{k}^{-i},{\bar{\theta }}_{k+1}^{-i},\ldots ,{\bar{\theta }}_{m}^{-i}\right]$. In this way, the auctioneer adopts a decision for allocation and requires all players to announce their opinion.

If everyone is satisfied, the auctioneer announces his/her decision to datacenter and finishes the game. Users execute their tasks and pay resource price. The algorithm of auctioneer and user procedure, along with more details, is presented in the following section. From the user’s view after task execution request, observers concentrate on analyzing the received message to understand his/her next move. When the auctioneer announces that a new auction has begun, the user calculates a new proposal amount and sends to the auctioneer. If a user receives a message containing parameters from alternate bids, he/she tries to estimate the next total bids, using Bayesian learning mechanism. And, in the end, when the user received the resource final price and the amount of his/her allocated share, he/she prepares for task execution and resource price update. This process has been demonstrated in algorithm 1. In the conducted simulation, the auction time was considered once in intervals of one hour. Upon starting of the auction, a message is sent to all users, containing a request for posting bid. After receiving all bids, the auctioneer informs users of the total Θ_*k*_. Simultaneously also, the auctioneer collects all bidding function parameters (including deadlines and price limit) from all users and adopts a rational decision and reaches a price limit. If the difference of $\sum h_{k}^{i}$ and Θ_*k*_ is more than a predefined threshold limit value, the auctioneer continuously rejects the price and requests new price (resource price amount $h_{k}^{i}$ is calculated by considering deadlines and price limits per user). When the equilibrium amount is achieved, the performed resource allocation is announced to everyone. This process has been demonstrated in algorithm 2.

**Algorithm 1 User i bidding algorithm**

1: submit tasks to auctioneer

2: if observer receives message of inform start then

3: add current auction

4: end if

5: if observer receives message of call for bids then

6: set $\left(e_{ik}^{1},\ldots ,e_{ik}^{m-1}\right)$ ← $e_{ik}^{m}$

7: send message of proposal to auctioneer

8: end if

9: if observer receives message of call for parameters then

10: inquiry historical price $\left(\theta_{1}^{-i},\ldots ,\theta_{k-1}^{-i}\right)$ ← $\theta_{k}^{-i}$

11: forecast future price $\left({\bar{\theta }}_{1}^{-i},\ldots ,{\bar{\theta }}_{k-1}^{-i}\right)$ ← ${\bar{\theta }}_{k}^{-i}$

12: send message of competitor's information to auctioneer

13:end if

14:if observer receives message of resource price then

15: (Θ_1_, …, Θ_*k*−1_) ← Θ_*k*_

16: send message of task execution to resource

17: delete current auction

18:end if

**Algorithm 2 Auctioneer allocation algorithm**

Require: N ≥ 2

1:initialize auctioneer

2:while auction k do

3: set bidders to auction k

4: broadcast message to call for bids

5: while bidder’s proposal arrives do

6: collect proposal message from bidder

7: end while

8: broadcast message to inform Θ_*k*_

9: while bidder’s parameter arrives do

10: collect parameter message from bidder

11: end while

12: while bidders disagree proportion do

13: for all cloud users do

14: build new bid function $h_{k}^{i}$

15: end for

16: $difference=\sum h_{k}^{i}-\Theta_{k}$

17: if *difference*> threshold then

18: $\Theta_{k}=\sum h_{k}^{i}$

19: else

20: exit

21: end if

22: update vector $\left[\theta_{1}^{-i},\ldots ,\theta_{k}^{-i},{\bar{\theta }}_{k+1}^{-i},\ldots ,{\bar{\theta }}_{m}^{-i}\right]$

23: end while

24: broadcast message to inform resource price

25: stop the current and wait for a new auction

26:end while

27:delete auctioneer

## Evaluation

For the evaluation of the proposed model, the Cloudsim simulation environment has been used. To compare the obtained results, first an implementation of algorithms was conducted based on the proposed auction by Zaman [69] and the proposed workload was produced in that article. In the proposed model by Zaman, several auctions have been suggested with different decision-making mechanisms according to which the author has concluded that his proposed model presents a better response than alternate auction-based economic methods and even with respect to real-time nature of Cloud environments, the proposed method will have the most optimal allocation. To better compare the proposed model in this research, which is based on auction and uses game theory mechanism, the simulation of the new model was done 275 times, involving 25 times model execution on prepared archives and calculation of the average achieved results in each of the workload archives. In the end, the 11 obtained results were compared for both models separately.

## Work Load Selection

Since for Cloud environments, a public workload has not yet been published, therefore, in this research, PWA workload (Parallel Workloads Archive) was used [70].This workload is a combination of a rich set of executed tasks on the world’ large databases. In this workload, over 26 archives exist of which 11 archives was chosen for this research; in Table 4, their specifications, including archive’s name, time duration for data collection from database, number of all executed tasks in that time interval and number of all processors, are given.

**Table 4:** Specifications of used workload.

Name of archive	Time interval	Number of tasks	Number of processors
ANL-Intrepid-2009	8 months	68,936	163,840
DAS2-fs0-2003	12 months	225,711	144
DAS2-fs1-2003	12 months	40,315	64
DAS2-fs2-2003	12 months	66,429	64
DAS2-fs3-2003	12 months	66,737	64
DAS2-fs4-2003	11 months	33,795	64
LLNL-Atlas-2006	8 months	42,725	9,216
LLNL-Thunder-2007	5 months	121,039	4,008
LLNL-uBGL-2006	7 months	112,611	2,048
LPC-EGEE-2004	9 months	234,889	140
SDSC-DS-2004	13 months	96,089	1,664

Archives are chosen so as to have diversity from time aspect and number of tasks and also processing power. A database with 64 to 163840 various processors has been used to be able to simulate on platforms with different specifications. These workloads are given based on SWF format [71]. In this format, information related to posted tasks to databases are presented in the form of 8 fields; in the simulation of this research, six characteristics have been used (Table 5).

**Table 5:** Available characteristics in workload.

Characteristic title	Usage in simulation
Job Number	Job name
Submit Time	Time of sending task to Cloud
Run Time	Time needed to execute task (rounded in hours)
Number of Allocated Processors	Number of processors requested
Average CPU Time Used	Average time of central processing unit used
User ID	Identity of user who posts the task (auction player)

Next, a number of other characteristics were added to this workload by researchers, which include the following:

- Number of virtual machines required in proportion to the demanded type of task: to calculate the number and type of virtual machines required for each task, we use equation $\rho_{j}=1-\frac{T_{j}^{CPU}}{T_{j}^{R}}$, in which $T_{j}^{CPU}$ is the amount of average CPU time used and $T_{j}^{R}$ is total time of task execution. Using the obtained amount, a record is performed for m types of virtual machines and, accordingly, we achieve the number of required virtual machines of m types.
- Task budget limit: to calculate each task’s budget amount, first, its speed is calculated from equation $S_{j}=P_{j}*\frac{T_{j}^{CPU}}{T_{j}^{R}}$, in which *P*_*j*_ is the number of used processors. Afterwards, the calculated speed is multiplied by a random number. This random number μ is fixed for each group of users. Thus, all users are categorized into three groups, basedon the UserID value, and a number is given to each group.
- Task deadline: since no option is considered in the available archives to determine deadline, in this research, we consider the deadline equivalent to 4 to 8 times the time required to execute the task and randomly assign it to each task.

## Auction execution

Following, for auction execution, four virtual machines VM1, VM2, VM3, VM4 are assumed. The value N equivalent to the number of users and M the number total available processors are deduced from each of archive files. We assume that the hypothesized virtual machines contain weights (1, 2, 3, 4), respectively. Namely that the processing power and resources VM2 are twice VM1. The number of users participating in each auction, is a function of total number of users of that workload. Since the auction is held every each hour, all users whose time sending task is smaller than the current time and their deadline is not over can participate in the auction whether or not previously participated in the auction (except for users who have been announced as winners in previous auctions). In order to detect the required number of virtual machines for each user, the set (C1, C2, C3) is assumed as communication ratios. As an example, the amount (0.05, 0.15, 0.25) is interpreted such that if a task has communication ratio 0.05, this task needs μ*P*_*j*_ number of VM1 instance in such a way that *P*_*j*_ is the number of processors required by user *u*_*j*_. The remaining required processors are also calculated with the same ratio among alternate instances of virtual machine. To assign users and identify the amount μ, UserID is used and users are divided into five categories (userid mod 5). The total number of virtual machines available in Cloud is initially a function of h vector which indicates the percentage of each of the instances compared with the whole virtual machines available. In this research, this vector has been assumed as (0.07, 0.13, 0.27, 0.53) and (0.25,0.25,0.25,0.25). in Table 6, the assumed values are demonstrated for the simulation of the model. The basic price of each of VMs has been considered based on prices listed on Amazon.

**Table 6:** Characteristics of model simulation [69].

Specification title	Explanations	Value
N	Total users	From workload
M	Total processors	From workload
T	Simulation times	From workload
μ	A parameter to obtain the number of VM instances from the number of processors	0.5, 0.75
h	VM distribution among their types	(0.25, 0.25, 0.25, 0.25), (0.07, 0.13, 0.27, 0.53)
C1,C2,C3	Task communication ratio	(0.05, 0.15, 0.25)
(C_I_, C_R_)	Unemployment time cost and time of executing a VM unit	(0.05, 0.1), (0.1, 0.25), (0.15,0.5)

### Analysis of results

**Fig 6:**
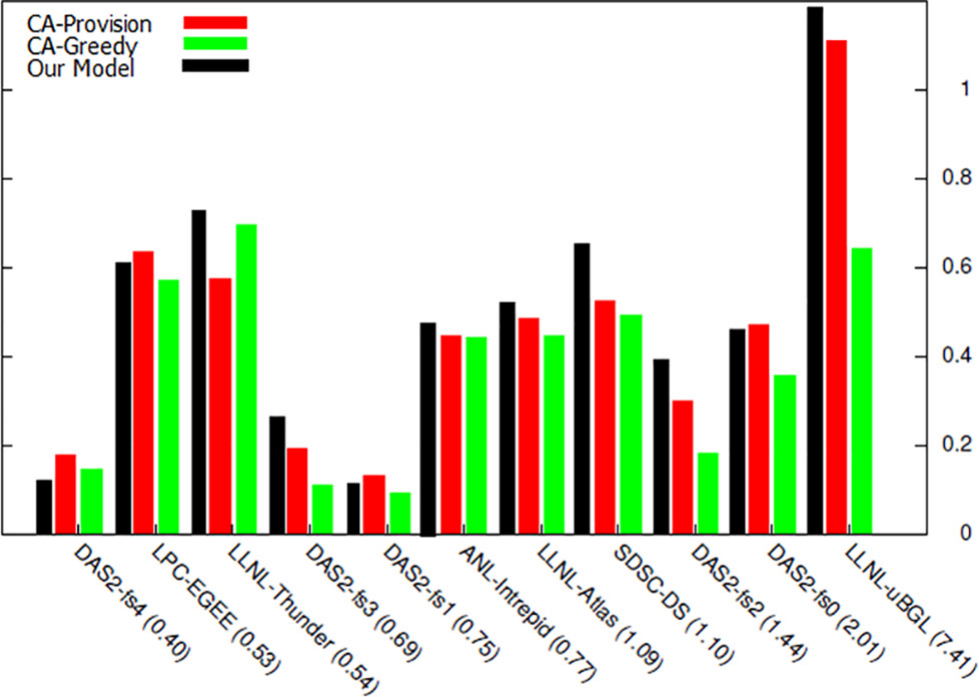
The resultant income from executing workload with normal distribution.

By executing the proposed model and analyzing the obtained results, a more accurate comparison can be performed among the methods under study. In the first stage, a comparison is conducted between the obtained income rate in the two proposed algorithms in [69] and this research model. In Fig 6, executing normal distribution of models has been shown on 11 selected archives. In workload number 1 and 5, the obtained income rate of the proposed model is less than the compared algorithms. The reason for this is the low number of available demands in this workload; since one of the bidding function calculation parameters is using the record of offered prices and as the number of demands becomes higher, the system will reach a more accurate forecasting with the passing of time; therefore, as observed, the proposed model has better response and greater income in most workloads compared with the other two algorithms.

**Fig 7:**
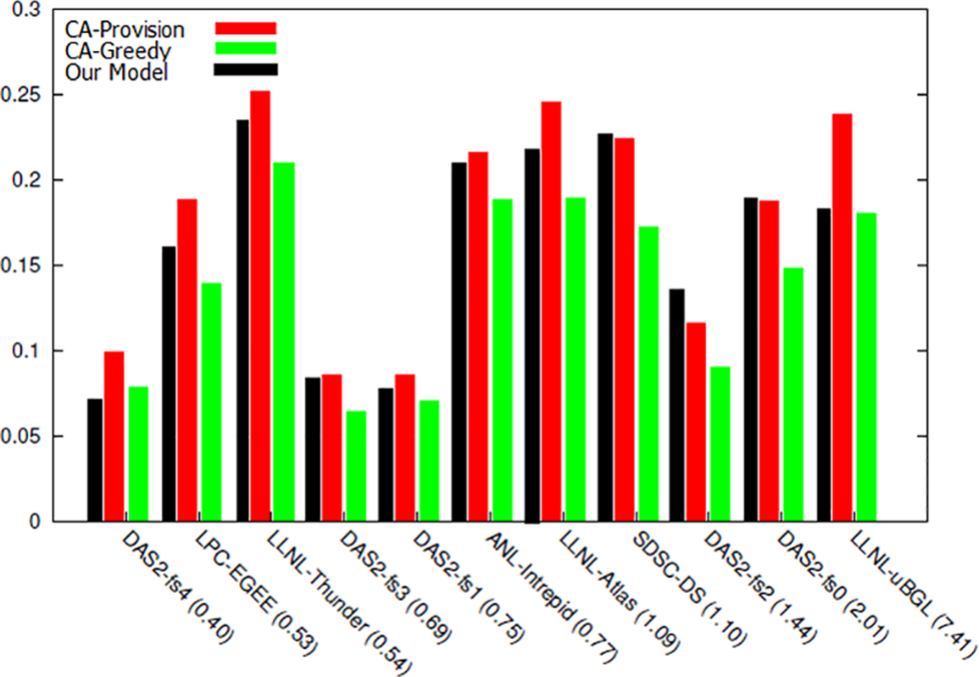
Cost of executing workload with normal distribution.

Next, based on the obtained outputs, the cost rate of executing the two previous proposed algorithms are displayed in Fig 7. As seen, the proposed model cost is either less than or equal to the two previous algorithms in most workloads, and the reason for this depends on the more rate of resource allocation performed in the proposed model.

**Fig 8:**
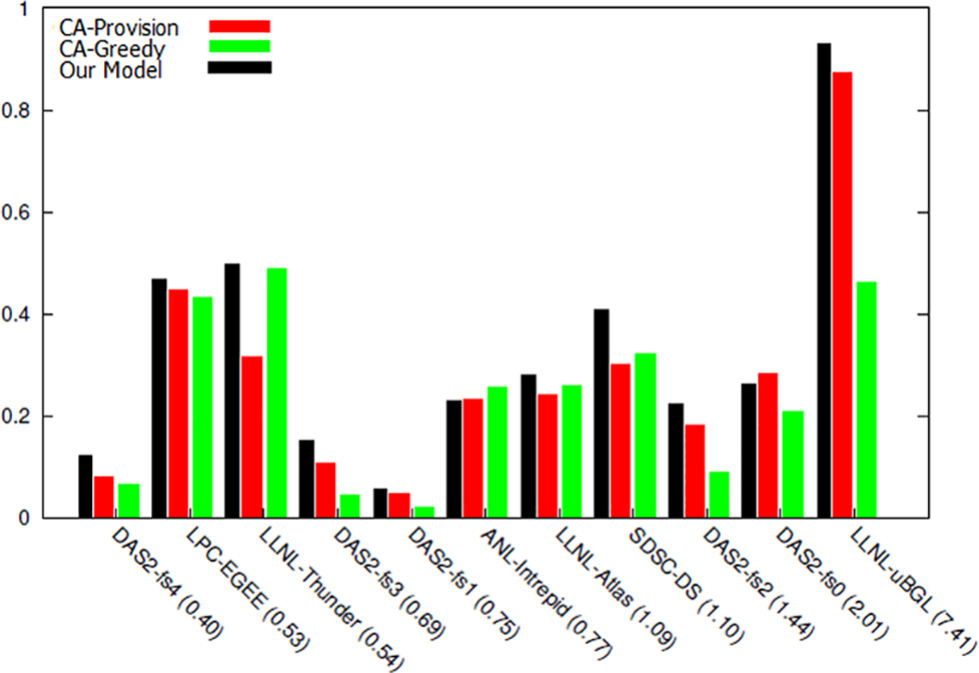
The resultant profit from workload with normal distribution.

In Fig 8, the rate of obtained final profit in each of workloads has been demonstrated by executing the three models. In 9 workloads, the rate of obtained profit is greater than both previous auctions and in 2 other workloads, most customers with sufficient deadline and enough budget to execute their task, have obtained their intended resource or resources. However, in the other two algorithms, two parameters of budget and time have not been included in resource allocation and have been chosen merely based on the highest winning proposed amount.

According to the obtained results in the comparison of the proposed model with other models, next, the effect of budget and time in choosing winner and the rate of resultant profit are investigated.

**Fig 9:**
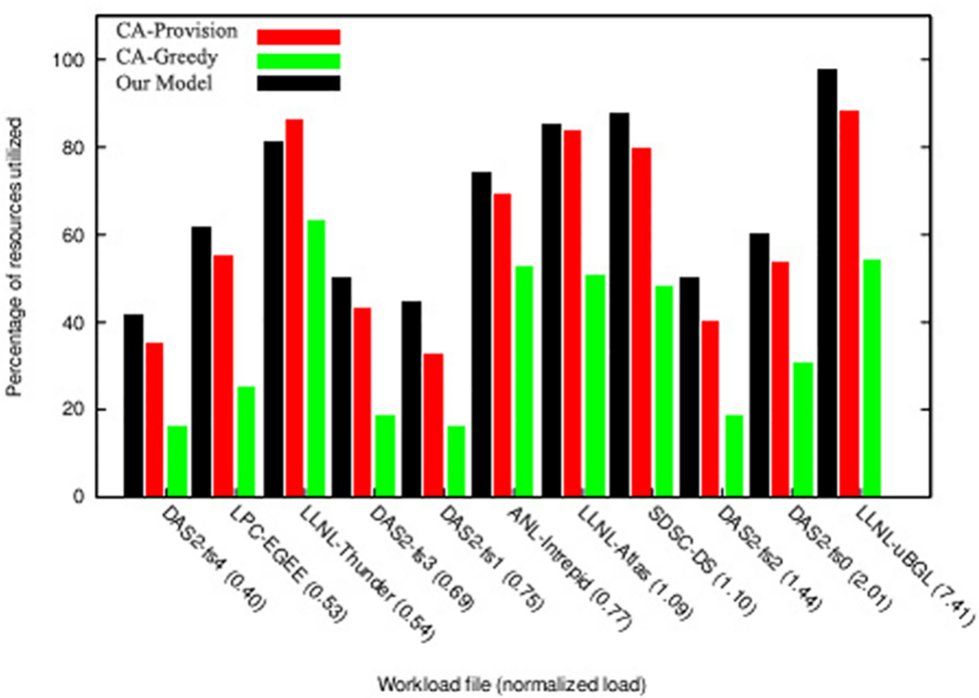
Percentage of resource utilization from workload with normal distribution.

**Fig 10:**
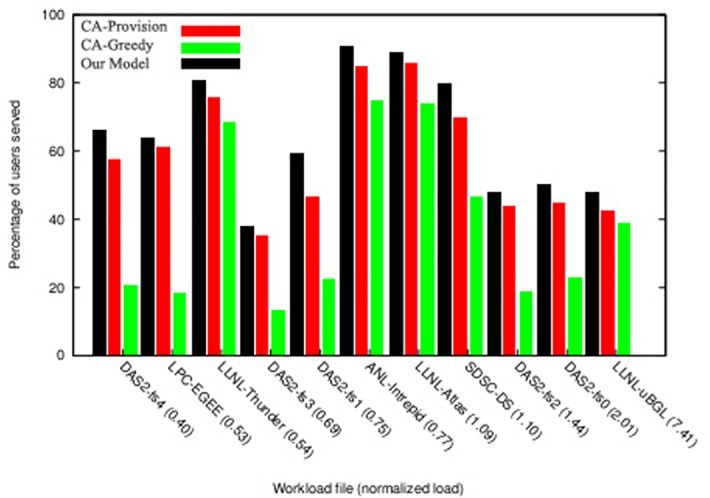
Percentage of user served in workload with normal distribution.

Fig 9 shows the extent of resources utilization in the proposed algorithm compared with other two algorithms. As can be seen in this figure, in the workloads in which resultant profit has been higher (Fig 8), the resource utilization has also been high and resources have been adequately utilized. Fig 10 shows the percentage of served users compared with total number of requests. This diagram shows that in the majority of workloads, the rate of response to users’ requests and the ability to perform their requests have been higher in the proposed algorithm compared with the other two algorithms. By analyzing the results of these two diagrams, it can be concluded that the mechanisms used in this study has a better performance in higher workloads compared with other economic methods, and also that this mechanism can achieve better results in cloud data centers that have a large number of users and repeated requests. The reason behind this claim is that while proposed mechanism provides equal opportunities for users to send their bids, it uses an objective function in which the parameters of budget constraint and deadline are considered and the price offered by the users ($h_{k}^{i}(\Theta_{k})$) and their deadlines move toward an equilibrium. The calculation of this function is based on the parameters announced by the users and the estimations of the auctioneer itself. On the other hand, users also utilize the bid function (*e*_*ik*_) with accordance to their budget constraints and deadline and then calculate and send the amount of their bid. This process causes the constraints of time and budget to be considered in both sides of the game, and the game ends when all players have offered their bids in accordance with their capabilities and needs and are satisfied with their performance and when no player has the desire to change his proposal; therefore, in this condition the Nash equilibrium is achieved.

**Fig 11:**
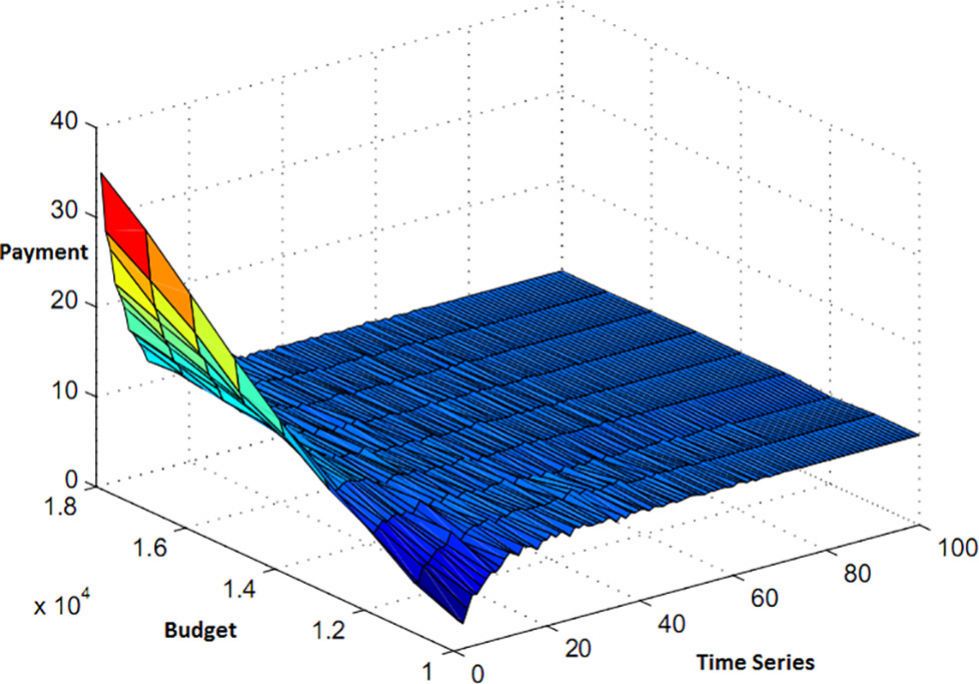
Convergence of bids in equilibrium mode.

In Fig 11, it has been demonstrated how prices alter during time. We conclude that applying budget has great effect on the initial equilibrium price since selfish but rational players constantly try to gain more profit than others. Nevertheless, in the mode of budget limitation, players become conservative and in order not to finish their budgets and to be able to pay for the execution of the remaining tasks, they act conservatively in the first stages; however, the equilibrium price is less than the average price. On the other hand, if players have sufficient capital, they are motivated to offer greater bids so as to be able to allocate a greater share of resources to themselves.

**Fig 12:**
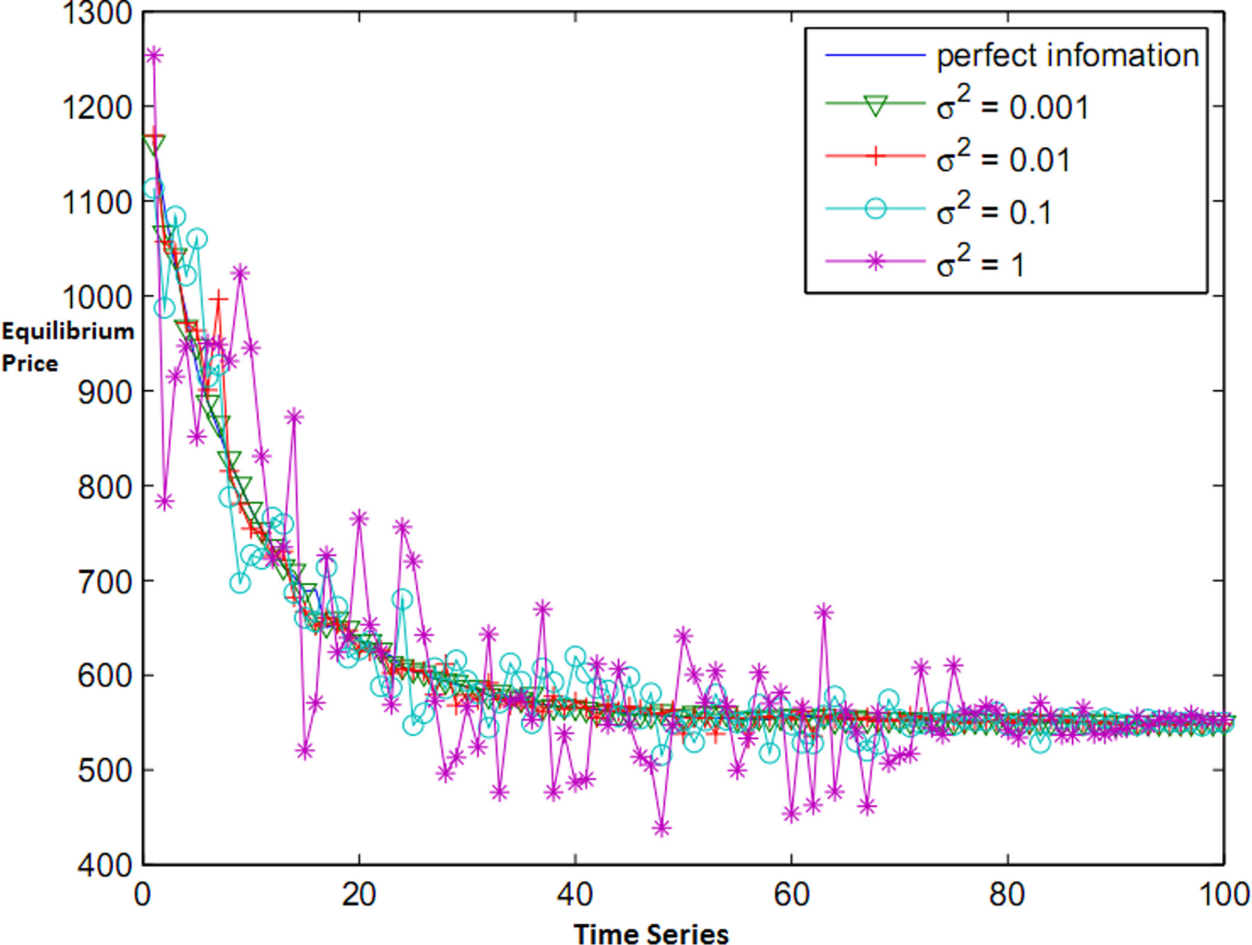
Resource price forecasting.

Next, the accuracy of forecasting Bayesian learning is evaluated in a mode where the cloud competitive environment is replete with uncertainty such as insufficient knowledge of the environment and posting tasks as real time. In Fig 12, price forecasting of resources in a dynamic game with incomplete information has been demonstrated.

When common knowledge is insufficient, the user tries to predict the others’ bids by using the published equilibrium prices. When the standard deviation is small, not more than 0.01, simulation works well. If users cause an unsustainable condition and propose random bids in a risky process, the accurate forecasting of prices becomes difficult. If the opposing information is iteratively learned, the practical results have shown that resource prices can still be convergent to equilibrium price in a step by step process.

It can be seen that LLNL-Thunder-2007 workload achieves the highest profit for cloud but it is also one the workloads that provide service to the greatest number of users. Therefore, in the following, we will further investigate the details of this workload and the behavior of the proposed model and other algorithms in this workload. Table 7 presents the comparison between the performances of different algorithms in this workload and the number of tasks that (considering their deadline) have been able to participate in the auction and also the tasks assigned to resources. This comparison shows that in naïve auction, a significant number of tasks can enter the auction but fewer numbers can get their desired resources. Other models also have been able to assign more than 80% of tasks to resources; but in the model proposed in this study, a high percentage of tasks receive service and the rate of agreement violation is very small.

**Table 7:** Summary of allocation rate for sample workload.

LLNL-Thunder-2007	Tasks	Auctions(Completed/Fails)		Allocated
CA-Greedy	121,039	6,542	114,497	102,680(85%)
CA-Provision	121,039	8,500	112,539	100,431(83%)
Naïve Auction	121,039	22,456	98,583	44,561(37%)
Our Model	121,039	154	120,885	120,784(99%)

## Conclusion

In this paper, a non-cooperative game is proposed to solve the problem of multi-user allocation in cloud scenarios. The scheduling model includes auction, bidding function, cost prediction and equilibrium point analysis. Here, an algorithm based on game theory is performed for user bidding, auctioneer pricing and share-bidding model in supplying resources in cloud framework simulator for scientific workflow tasks. The results demonstrated that resource allocation during a non-cooperative game between users achieves Nash equilibrium point even when our common knowledge of the environment is insufficient. In this paper, it was demonstrated that forecasting Bayesian learning has the best and more sustainable efficiency and can help the auctioneer to reach resource equilibrium price. In the proposed model, in order to execute tasks with workflow at the level of platform as a service, each player had limitations in budget and deadline for his/her intended task execution; therefore, a method was proposed so as to be able to reach the game condition to an equilibrium point, that is Nash equilibrium point by offering an objective function for the estimation of the bidding amount by the player in the auction and also offering a method to choose a suitable price by the auctioneer. Model simulation showed that despite competition among users and presence of users with various financial capability and deadlines in the execution of their demands, the system achieves a sustainable mode after executing a few stages and the proposed bidding function prevents from reaching impasse. Comparing the proposed model with some auction-oriented methods revealed that in the proposed model in this research, the profit rate resulting from allocations are greater compared with other methods and more users obtain their intended resource.

## References

[1] Armbrust M, Fox A, Griffith R. "Above the clouds: A berkeley view of cloud computing," University of California, Berkeley, 2009.

[2] Buyya R, Abramson D, Giddy J, Stockinger H. "Economic models for resource management and scheduling in grid computing," Concurrency and Computation: Practice and Experience, vol. 14, p. 1507–1542, 2002.

[3] Fan Q, Wu Q, Magoulés F, Xiong N, Vasilakos AV, He Y. "Game and balance multicast architecture algorithms for sensor grid," Sensors, vol. 9, no. 9, p. 7177–7202, 2009 doi: 10.3390/s90907177 22399992 10.3390/s90907177 PMC3290501

[4] Gibbons R. A Primer in Game Theory, Pearson Higher Education, 1992.

[5] Sandholm TW. "Multiagent systems: a modern approach to distributed artificial intelligence," Distributed rational decision making, vol. 37, p. 201–258, 1999.

[6] Wei G, Vasilakos A, Zheng Y, Xiong N. "A game-theoretic method of fair resource allocation for cloud computing services," The Journal of Supercomputing, vol. 54, pp. 1–18, 2009.

[7] Kwok YK, Song S, Hwang K. "Selfish grid computing: Game-theoretic modeling and nash performance results," in In Proceedings of International Symposium on Cluster Computing and the Grid, 2005.

[8] Galstyan A, Kolar S, Lerman K. "Resource allocation games with changing resource ca-pacities," in In Proceedings of the International Conference on Autonomous Agents and Multi-Agent Systems, 2003.

[9] Bredin J, Kotz D, Rus D, Maheswaran RT, Imer C, Basar T. "Computational markets to regulate mobile-agent systems," Autonomous Agents and Multi-Agent Systems, vol. 6, p. 235–263, 2003.

[10] Maheswaran RT, Basar T. "Nash equilibrium and decentralized negotiation in auctioning divisible resources," Group Decision and Negotiation, vol. 12, p. 361–395, 2003.

[11] Khan S, Ahmad I. "Non-cooperative, semi-cooperative, and cooperative games-based grid resource allocation," in Parallel and Distributed Processing Symposium, 2006.

[12] An B, Miao C, Shen Z. "Market based resource allocation with incomplete information," in In Proceedings of the 20th International Joint Conference on Artifical Intelligence, 2007.

[13] Teng F, Magoulès F. "A new game theoretical resource allocation algorithm for cloud computing," in In Proceedings of the 5th international conference on Advances in Grid and Pervasive Computing (GPC'10), 2010.

[14] Garg S, Buyya R. "Market-Orient ed Resource Management and Scheduling: A Taxonomy and Survey," Cooperative Networking, pp. 277–306, 2011.

[15] Sutherland E. "A futures market in computer time," Communications of the ACM, vol. 11, no. 6, p. 449–451, 1968.

[16] Gagliano RA, Fraser MD, Schaefer ME. "Auction allocation of computing resources," Communications of the ACM, vol. 38, no. 6, p. 88–102, 1995.

[17] Gibney MA, Jennings NR, Vriend NJ, Griffiths JM. "Market-based call routing in telecommunications networks using adaptive pricing and real bidding," in In Proceedings of the Third International Workshop on Intelligent Agents for Telecommunication Applications, 1999.

[18] Anselmi J, Ardagna D, Passacantando M. "Generalized Nash equilibria for SaaS/PaaS clouds," Eur J Oper Res.

[19] Chun BN, Culler DE. "User-centric performance analysis of market-based cluster batch schedulers," in In Proceedings of the 2nd IEEE/ACM International Symposium on Cluster Computing and the Grid, Washington, DC, USA, 2002.

[20] TM Nguyen H, Magoulés F. "Autonomic data management system in grid environment," Journal of Algorithms & Computational Technology, vol. 3, p. 155–177, 2009.

[21] Stuer G, Vanmechelen K, Broeckhove J. "A commodity market algorithm for pricing substitutable grid resources," Future Generation Computer Systems, vol. 23, no. 5, p. 688–701, 2007.

[22] Stratford N, Mortier R. "An economic approach to adaptive resource management," in In Proceedings of the 7th Workshop on Hot Topics in Operating Systems, 1999.

[23] Ozturan C. "Resource bartering in data grids," Science of Computer Programming, vol. 12, no. 3, p. 155–168, 2004.

[24] Wolski R, Plank JS, Brevik J, Bryan T. "Analyzing market-based resource allocation strategies for the computational grid," Intl. J. of High Performance Comp Appl., vol. 15, no. 3, p. 258–281, 2001.

[25] Gomoluch J, Schroeder M. "Market-based resource allocation for grid computing:A model and simulation," in In Proc. 1st International Workshop on Middleware for Grid Computing, 2003.

[26] Das A, Grosu D. "Combinatorial auction-based protocols for resource allocation in grids," in In Proc. 19th International Parallel and Distributed Processing Symposium, 6th Workshop on Parallel and Distributed Scientific and Engineering Computing, 2005.

[27] Dash RK, Vytelingum P, Rogers A, David E, Jennings NR. "Market-based task allocation mechanisms for limited-capacity suppliers," IEEE Transactions on Systems, Man and Cybernetics–Part A: Systems and Humans, vol. 37, no. 3, p. 391–405, 2007.

[28] Grosu D. "AGORA: An architecture for strategy proof computing in grids," in In Proc. 3rd International Symposium on Parallel and Distributed Computing, 2004.

[29] Wolski R, Plank JS, Brevik J, Bryan T. "G-commerce: Market formulations controlling resource allocation on the computational grid," in In Proceedings of International Parallel and Distributed Processing Symposium, 2001.

[30] Stanoevska-Slabeva K, Parrilli M, Thanos G. "Beingrid: Development of business models for the grid industry," in In Proceedings of International Workshop on Grid Economics and Business Models, 2008.

[31] Risch M, Altmann J, Guo L, Fleming A, Courcoubetis C. "The GridEcon platform: A business scenario testbed for commercial cloud services," in In Proc. Workshop on Grid Economics and Business Models, 2009.

[32] Wang H, Jing Q, Chen R, He B, Qian Z, Zhou L. "Distributed systems meet economics: Pricing in the cloud," in In Proc. 2nd USENIX Workshop on Hot Topics in Cloud Computing, 2010.

[33] Walker E, Brisken W, Romney J. "To lease or not to lease from storage clouds," IEEE Computer, vol. 43, no. 4, p. 44–50, 2010.

[34] Li A, Yang X, Kandula S, Zhang M. "CloudCmp: Shopping for a cloud made easy," in In Proc. 2nd USENIX Workshop on Hot Topics in Cloud Computing, 2010.

[35] Buyya R, Ranjan R, Calheiros RN. "InterCloud: Utility-oriented federation of cloud computing environments for scaling of application services," in In Proc. 10th International Conference on Algorithms and Architectures for Parallel Processing, 2010.

[36] Altmann J, Courcoubetis C, Stamoulis GD, Dramitinos M, Rayna T, Risch M, et al. "GridEcon: A market place for computing resources," in In Proc. Workshop on Grid Economics and Business Models, 2008.

[37] Lin W-Y, Lin G-Y, Wei H-Y. "Dynamic auction mechanism for cloud resource allocation," in In Proc. 2010 10th IEEE/ACM International Conference on Cluster, Cloud and Grid Computing, 2010.

[38] Chohan N, Castillo C, Spreitzer M, Steinder M, Tantawi A, Krintz C. "See spot run: Using spot instances for MapReduce workflows," in In Proc. 2nd USENIX Workshop on Hot Topics in Cloud Computing, 2010.

[39] Campos-Nanez E, Fabra N, Garcia A. "Dynamic auctions for on-demand services," IEEE Transactions on Systems, Man and Cybernetics–Part A: Systems and Humans, vol. 37, no. 6, p. 878–886, 2007.

[40] Quiroz A, Kim H, Parashar M, Gnanasambandam N, Sharma N. "Towards autonomic workload provisioning for enterprise grids and clouds," in In Proc. 10th IEEE/ACM International Conference on Grid Computing, 2009.

[41] Van HN, Tran FD, Menaud J-M. "Autonomic virtual resource management for service hosting platforms," in In Proc. ICSE Workshop on Software Engineering Challenges in Cloud Computing, 2009.

[42] Vecchiola C, Calheiros RN, Karunamoorthy D, Buyya R. "Deadline-driven provisioning of resources fro scientific applications in hybrid clouds with Aneka," Future Generation Computer Systems, vol. 28, p. 58–65, 2012.

[43] Kansal A, Zhao F, Liu J, Kothari N, Bhattacharya AA. "Virtual machine power metering and provisioning," in In Proc. 1st ACM Symposium on Cloud computing, 2010.

[44] Meng X, Isci C, Kephart J, Zhang L, Bouillet E, Pendarakis D. "Efficient resource provisioning in compute clouds via vm multiplexing," in In Proc. 7th international conference on Autonomic computing, 2010.

[45] Chen W, Qiao X, Wei J, Huang T. "A profit-aware virtual machine deployment optimization framework for cloud platform providers," in In Proc. 5th IEEE International Conference on Cloud Computing, 2012.

[46] Ghosh R, Naik VK. "Biting of safely more than you can chew: Predictive analytics for resource over-commit in IaaS cloud," in In Proc. 5th IEEE International Conference on Cloud Computing, 2012.

[47] Lynar TM, Herbert RD, Simon S. "Auction resource allocation mechanisms in grids of heterogeneous computers," WSEAS Transactions on Computers, vol. 8, no. 10, p. 1671–1680, 2009.

[48] Buyya R, Ranjan R, Calheiros RN. "Modeling and simulation of scalable cloud computing environments and the cloudsim toolkit: Challenges and opportunities," in In Proceedings of the 7th High Performance Computing and Simulation Conference, 2009.

[49] Sequeira S, Karthikeyan P. "A survey on auction based resource allocation in cloud computing," International Journal of Research in Computer Applications and Robotics, vol. 1, no. 9, pp. 96–102, 2013.

[50] Khanna G, Beaty K, Kar G, Kochut A. "Application performance management in virtualized server environments," in in Proc. of the IEEE Network Ops.and Mgmt, 2006.

[51] Steinder M, Whalley I, Carrera D, Gaweda I, Chess D. "Server virtualization in autonomic management of heterogeneous workloads," in in Proc. of the IEEE Sym. on Integrated Network Management, 2007.

[52] Kephart J, Chan H, Levine D, Tesauro G, Rawson F, Le-furgy C. "Coordinating multiple autonomic managers to achieve specified power-performance tradeoffs," in in Proc. IEEE Intl. Conf. on Autonomic Computing (ICAC), 2007.

[53] Ranganathan P, Leech P, Irwin D, Chase J. "Ensemble-level power management for dense blade servers," in in Proc. of the IEEE Sym. on Computer Architecture, 2006.

[54] Pinheiro E, Bianchini R, Heath T. Dynamic Cluster Reconfiguration for Power and Performance, Kluwer Academic Publishers, 2003.

[55] Abdelzaher T, Shin K, Bhatti N. "Performance Guarantees for Web Server End-Systems: A Control-Theoretical Approach," IEEE Trans. Parallel and Distributed Systems, vol. 13, no. 1, pp. 80–96, 2002.

[56] Kusic D, Kandasamy N. "Risk-Aware Limited Lookahead Control for Dynamic Resource Provisioning in Enterprise Computing Systems," in Int’l Conf. Autonomic Computing, 2006.

[57] Kusic D, Kephart J, Kandasamy N, Jiang G. "Power and Performance Management of Virtualized Computing Environments via Lookahead Control," in Int’l Conf. Autonomic Computing, 2008.

[58] Qin W, Wang Q. "Modeling and Control Design for Performance Management of Web Servers via an LPV Approach," IEEE Trans. Control Systems Technology, vol. 15, no. 2, pp. 259–275, 2007.

[59] Raghavendra R, Ranganathan P, Talwar V, Wang Z, Zhu X. "No ‘Power’ Struggles: Coordinated Multi-Level Power Management for the Data Center," SIGARCH Computer Architecture News, vol. 36, no. 1, pp. 48–59, 2013.

[60] Joita L, Rana OF, Freitag F, Chao I, Chacin P, Navarro L, et al "A catallactic market for data mining services," Future Generation Computer Systems, vol. 23, no. 1, p. 146–153, 2007.

[61] Garg SK, Venugopal S, Broberg J, Buyya R. "Double auction-inspired meta-scheduling of parallel applications on global grids," Journal of Parallel and Distributed Computing, 2013.

[62] Risch M, Altmann J, Guo L, Fleming A, Courcoubetis C. "The gridecon platform: A business scenario testbed for commercial cloud services," Seoul National University, Technology Management, Economics and Policy Program, 2009.

[63] Ardagna D, Panicucci B, Passcantando M. "Generalized Nash Equilibria for the Service Provisioning Problem in Cloud Systems," IEEE Transactions on Services Computing, vol. 6, no. 4, pp. 429–442, 2013.

[64] Ding S, Wang J, Ruan W, Xia C. Inferring to individual diversity promotes the cooperation in the spatial prisoner’s dilemma game. Chaos, Solitons & Fractals, 71: 91–99, 2015.

[65] Ding S, Yang S, Zhang Y, Liang C, Xia C. Combining QoS prediction and customer satisfaction estimation to solve cloud service trustworthiness evaluation problems. KNOWLEDGE-BASED SYSTEMS 56: 216–115, 2014.

[66] Ding S, Xia C, Zhoa K, Yang S, Shang J. Decision support for personalized cloud service selection through multi-attribute trustworthiness evaluation, PLoS ONE 9 (6): e107821, 2014. 10.1371/journal.pone.0097762 PMC4074036 24972237

[67] Ding S, Xia C, Cai Q, Zhou K, Yang S. QoS-aware resource matching and recommendation for cloud computing systems, Applied Mathematics and Computation 247: 941–950, 2014.

[68] Gintis H. Game Theory Evolving: A Problem-Centered Introduction to Modeling Strategic Interaction (Second Edition). Princeton University Press, Princeton, 2009.

[69] Zaman S, Grosu D. "A Combinatorial Auction-Based Mechanism for Dynamic VM Provisioning and Allocation in Clouds," Cloud Computing, IEEE Transactions on, vol. 1, no. 2, pp. 129–141, 2013.

[70] Feitelson D. "Parallel Workloads Archives: Logs.," [Online]. Available: http://www.cs.huji.ac.il/labs/parallel/workload/logs.html.

[71] Feitelson D. "Parallel Workloads Archives: Standard Workload Format.," [Online]. Available: http://www.cs.huji.ac.il/labs/parallel/workload/swf.html.

